# Diagnostic Accuracy of Cross-Polarization OCT and OCT-Elastography for Differentiation of Breast Cancer Subtypes: Comparative Study

**DOI:** 10.3390/diagnostics10120994

**Published:** 2020-11-24

**Authors:** Ekaterina V. Gubarkova, Elena B. Kiseleva, Marina A. Sirotkina, Dmitry A. Vorontsov, Ksenia A. Achkasova, Sergey S. Kuznetsov, Konstantin S. Yashin, Aleksander L. Matveyev, Aleksander A. Sovetsky, Lev A. Matveev, Anton A. Plekhanov, Alexey Y. Vorontsov, Vladimir Y. Zaitsev, Natalia D. Gladkova

**Affiliations:** 1 Institute of Experimental Oncology and Biomedical Technologies, Privolzhsky Research Medical University, 603950 Nizhny Novgorod, Russia; kiseleva84@gmail.com (E.B.K.); sirotkina_m@mail.ru (M.A.S.); achkasova.k@bk.ru (K.A.A.); strike_gor@mail.ru (A.A.P.); natalia.gladkova@gmail.com (N.D.G.); 2 Department of Oncology, Nizhny Novgorod Regional Oncologic Hospital, 603126 Nizhny Novgorod, Russia; dr.vorontsovdmitriy@rambler.ru (D.A.V.); doctorvorontsov@mail.ru (A.Y.V.); 3 Department of Pathology, Privolzhsky Research Medical University, 603950 Nizhny Novgorod, Russia; zunek@mail.ru; 4 University Clinic, Privolzhsky Research Medical University, 603950 Nizhny Novgorod, Russia; jashinmed@gmail.com; 5 Laboratory of Multidimensional Signal Processing, Institute of Applied Physics of the Russian Academy of Sciences, 603950 Nizhny Novgorod, Russia; matveyev@appl.sci-nnov.ru; 6 Laboratory of Wave Methods for Studying Structurally Inhomogeneous Media, Institute of Applied Physics of the Russian Academy of Sciences, 603950 Nizhny Novgorod, Russia; alex.sovetsky@mail.ru (A.A.S.); lionnn52rus@mail.ru (L.A.M.); vyuzai@ipfran.ru (V.Y.Z.)

**Keywords:** breast cancer, cross-polarization optical coherence tomography (CP-OCT), compressional optical coherence elastography (C-OCE), image assessment

## Abstract

The possibility to assess molecular-biological and morphological features of particular breast cancer types can improve the precision of resection margin detection and enable accurate determining of the tumor aggressiveness, which is important for treatment selection. To enable reliable differentiation of breast-cancer subtypes and evaluation of resection margin, without performing conventional histological procedures, here we apply cross-polarization optical coherence tomography (CP-OCT) and compare it with a novel variant of compressional optical coherence elastography (C-OCE) in terms of the diagnostic accuracy (Ac) with histological verification. The study used 70 excised breast cancer specimens with different morphological structure and molecular status (Luminal A, Luminal B, Her2/Neo+, non-luminal and triple-negative cancer). Our first aim was to formulate convenient criteria of visual assessment of CP-OCT and C-OCE images intended (i) to differentiate tumorous and non-tumorous tissues and (ii) to enable more precise differentiation among different malignant states. We identified such criteria based on the presence of heterogeneities and characteristics of signal attenuation in CP-OCT images, as well as the presence of inclusions/mosaic structures combined with visually feasible assessment of several stiffness grades in C-OCE images. Secondly, we performed a blinded reader study of the Ac of C-OCE versus CP-OCT, for delineation of tumorous versus non-tumorous tissues followed by identification of breast cancer subtypes. For tumor detection, C-OCE showed higher specificity than CP-OCT (97.5% versus 93.3%) and higher Ac (96.0 versus 92.4%). For the first time, the Ac of C-OCE and CP-OCT were evaluated for differentiation between non-invasive and invasive breast cancer (90.4% and 82.5%, respectively). Furthermore, for invasive cancers, the difference between invasive but low-aggressive and highly-aggressive subtypes can be detected. For differentiation between non-tumorous tissue and low-aggressive breast-cancer subtypes, Ac was 95.7% for C-OCE and 88.1% for CP-OCT. For differentiation between non-tumorous tissue and highly-aggressive breast cancers, Ac was found to be 98.3% for C-OCE and 97.2% for CP-OCT. In all cases C-OCE showed better diagnostic parameters independently of the tumor type. These findings confirm the high potential of OCT-based examinations for rapid and accurate diagnostics during breast conservation surgery.

## 1. Introduction

Intraoperative detection of breast malignancy margins would allow minimization of the risk of tumor recurrence in patients undergoing breast conservation surgery (BCS). Intraoperative pathological estimation can be performed through frozen section analysis and imprint cytology [1]; however, these techniques are characterized by several restrictions such as resource intensity, sampling only a small percentage of the surgical margins and limited efficacy, especially for ductal carcinoma in situ (DCIS) [2]. Consequently, these methods have not been widely adopted [3]. Fluorescent techniques that utilize molecular contrast, potentially affording surgeons to visualize tumor in the cavity, are currently in the development [4,5]. Ultrasound elastography has been developed for a number of applications, and specifically for preoperative diagnosis of breast lesions [6,7,8]. However, its relatively low spatial resolution makes it inappropriate to use this method for intraoperative tumor margin assessment.

Optical coherence tomography (OCT) presents a very promising method for surgical tasks solving due to the clear benefits of this method such as: safety (using a near infrared light source does not risk tissue damage); accuracy (high resolution ~10–15 micron); there being no need for contrast agents; and the short duration of image attainment. OCT can be used both in the resected specimen of tumor and in the surgical cavity. OCT can be added to biopsy needle probes and can be used to guide correct sampling of tumor biopsies [9,10,11]. OCT is a promising method for intraoperative guidance during the resection of breast cancer and for identifying positive margins in specimens from BCS [12,13,14,15]. Recently, OCT has been proposed for intraoperative use in distinguishing tumorous and non-tumorous tissues using handheld probes [16,17]. To overcome the limited imaging depth ~2 mm typical for OCT (which usually requires sufficiently close approaching of the OCT probe towards the studied tissue), utilization of endoscopic and/or needle OCT probes are considered [11]. Moreover, it is known that structural OCT scans exhibit low contrast between tumor and uninvolved dense stromal tissue, which makes it challenging to accurately assess margin status [18]. In view of this, improvements in several aspects of OCT attract much attention, in particular, the development of functional OCT extensions based on polarization effects and stiffness analysis as considered below.

Based on the birefringence of the tissue structure, polarization-sensitive (PS) OCT provides advanced imaging of collagen fibers in the breast tissue and enhances intraoperative differentiation of breast cancer [19,20,21]. Stroma state assessment is fundamentally important, because tumor collagen matrix plays a crucial role in breast cancer invasion and metastatic spreading [22]. Several studies have developed quantitative diagnosis algorithms for intraoperative assessing breast cancer margins and validated them against OCT, both alone and in combination with other modalities [23]. Cross-polarization OCT (CP-OCT) is a variant of PS OCT that allows imaging of the initial polarization state changes due to both birefringence and cross-scattering in biological tissues [24]. Only orthogonally polarized backscattered light, which is mutually coherent with the incident wave, contributes to the cross-polarized (CP) OCT image. CP-OCT is a promising method for differentiating tumorous from non-tumorous tissues in human breast tissues [25], human brain tissues [26,27], as well as for diagnosis of bladder cancer [28,29,30,31]. OCT can also measure attenuation, which can be helpful for improving contrast of breast imaging research [32,33].

Attention to the problem of determining tissue stiffness (elastographic mapping) by optical coherence elastography (OCE) methods has been increasing in recent years [34,35,36,37]. Sufficiently high resolution of quantitative stiffness maps enabled by compressional OCE opened the possibility to perform morphological segmentations of tumor tissue constituents very similar to morphological segmentation of conventional histological images [25,38,39]. In these studies of experimental tumor models on animals, this technique allowed in vivo monitoring of morphological variation in tumor tissue during tumor growth and response to therapies. In studies [25,40,41,42], application of compressional OCE (C-OCE) for characterization of mechanical properties of excised human breast cancer specimens was demonstrated. New possibilities for intraoperative assessment of the breast cancer borders by means of optical coherence micro-elastography (OCME) were reported in a recent study [43,44]. It has been demonstrated that OCME provides additional contrast of tumor compared to OCT alone. Additionally, the potential of OCME images for evaluation of tumor margins in specimens excised during BCS was demonstrated in [41,43]. In our previous paper it was shown that CP-OCT and C-OCE can be helpful in breast cancer margin identification, as well as for grading breast cancer subtypes [25].

For more accurate evaluation of the resection margin, it is advantageous to take into account the genetic heterogeneity of breast cancer, as well as the variety of molecular-biological and morphological features influencing prognosis of the disease course (degree of aggressiveness) and treatment selection [45,46]. Indeed, it was demonstrated that probability of tumor recurrence mainly depends on molecular-biological characteristic of the tumor [47,48], while an increase in the size of the removed tissue, free of cancer cells, is not associated with a decrease in the recurrence rate [49].

Various molecular-biological and morphological features of breast cancer are anticipated to differently influence the polarizing and elastic tumor and peritumoral tissue qualities. This stimulates interest in evaluation of the clinical potential of polarization-sensitive and elastographic OCT techniques for determining breast cancer subtypes (malignancy grade) and improving tumor boundary detection based on the ability of these methods to identify different tumor subtypes. In this way, surgeons are expected to be provided with essential information that can improve reliability of the positive resection margin detection during BCS, at least for some breast cancer subtypes.

The goals of this research are (1) to define the visual assessment criteria required for the CP-OCT and C-OCE images in order to enable differentiation among various breast cancer subtypes; (2) to determine the diagnostic accuracy (sensitivity and specificity) of C-OCE in comparison with CP-OCT, for delineation of tumorous and non-tumorous breast tissues and subsequent identification of breast cancer subtypes in a blinded reader study.

## 2. Materials and Methods

### 2.1. Human Breast Specimens

This study was approved by the institutional review board of the Privolzhsky Research Medical University (Protocol #10 from 28 September 2018). All of the patients included in the study provided written informed consent. A total of 70 breast tumor tissue specimens were taken from 50 patients post partial (*n* = 35) or complete (*n* = 15) mastectomy with different diagnosis (Table 1). To minimize the effect tissue degrading, the excised specimens were immediately placed in gauze saturated with phosphate buffer and closed to prevent dehydration. CP-OCT and C-OCE images of the fresh, un-fixed breast tissue were acquired within 2 h after surgical excision. The studies were done on specimens with sizes from 0.5 × 1 cm to 1 × 2 cm. Specimens were taken from central zone of tumors for diagnostics of breast cancer subtypes and in the peritumoral area for visualization of normal (non-tumorous) breast tissue. A special motorized table for convenient positioning the specimen under the OCT probe was used. The entire CP-OCT and C-OCE study of each specimen was no longer than 20 min (including preliminary sample preparation and orientation).

### 2.2. Multimodal OCT Device

This study used a common path spectral domain multimodal OCT system with a central wavelength of 1310 nm and spectral width of 100 nm, with an axial resolution of 10 µm, lateral resolution is 15 μm, a scanning depth of 2 mm in air, a scanning speed of 20,000 A-scans per second. The OCT-system acquired 3D blocks of OCT data, 2 mm in depth (in air) over 2.4 × 2.4 mm^2^ area and 2D lateral scanning with a similar field of view were acquired in 26 s. The CP-OCT and C-OCE images were generated in real time during the acquisition process. For living tissues, real-time angiographic imaging was also possible by processing the same data [50]. For the described OCT studies, the total scanning time along a 1–2 cm trajectory on a biopsy sample was 3–5 min depending on the number of stitched images.

Structural 2D (cross-sectional images) CP-OCT images were constructed in two virtual channels, one of which was co-polarized with the incident polarization (co-polarization channel) and the other one was orthogonal (cross-polarization channel) to the incident polarization, respectively [24]. CP-OCT aims to obtain the information contained in the cross-polarization channel, which allows one to form cross-polarization images caused by birefringence of the tissue from optically anisotropic structures (evaluate the state of connective tissue component), as well as due to contribution of coherent cross-polarization backscattering on non-spherical particles and particles with dimensions much larger than the wavelength. In view of low informativity of the co-polarization images (as found in previous studies [25]), only cross-polarization images were used for diagnostic conclusions in this study.

An advanced variant of phase-sensitive compression OCE [37,39,51,52,53,54,55] was used to visualize inter-frame strains in the tissue and subsequently map the Young modulus. The probe was slightly pressed onto the studied sample surface, and strain distribution in the probe vicinity was reconstructed. Strain mapping was based on estimation of axial gradients of interframe phase variations of the OCT signal using the “vector” method [51,53]. The name “vector” is due to the fact that, without explicitly singling out amplitude and phase, the complex-valued OCT signals in this method are considered as vectors in the complex plane, and the phase is singled out at the very last step of the processing. Such vector representation allows one to perform flexibly-tuned amplitude-weighted averaging over the processing-window area. As a result, noisy contributions of small-amplitude pixels and, at the same time, especially strong phase errors (by ~π rad.) are very efficiently suppressed. This allows obtaining strain maps with fairly high quality even without periodic averaging (which is very important for the one-directional single-step loading of the tissue used in the described studies). In addition to the exceptionally high tolerance to various measurement noises, the vector method is very efficient computationally, so that the elastographic processing of the acquired sequence of several hundreds of OCT scans requires ~5–10 s using a “typical” PC without the necessity of GPU computations.

Another important point is that the estimated interframe phase-variation gradient is averaged over a processing window, the dimensions of which being the main factor determining the resolution of the resultant OCE scans. For a rectangular processing window with comparable axial and lateral sizes, the resolution in strain maps is also comparable in these directions and corresponds to ~½ of the window size. For the described system, the window size was ~90–100 µm, which defined the strain-mapping resolution ~45–50 µm. Such a window size was chosen empirically as a compromise between worse quality of the OCE-images for smaller windows (because of insufficiently averaged noise) and too-strong smoothing of spatial inhomogeneities for larger windows.

The next important point is quantification of the tissue Young’s modulus, to enable which a reference silicone layer with preliminary calibrated stiffness (with the Young’s modulus in the range 50–100 kPa) was used as described in [42,54,55,56]. Of key importance in the used variant of C-OCE technique is that all OCE images are formed using a pre-selected pressure level (4 kPa in the described study) standardized over the entire image area, despite the fact that for real OCE scans, the local pressure over the lateral coordinate usually varies several times because of the non-ideally planar boundary of the sample, its mechanical inhomogeneity, etc. The pressure standardization technique is based on the usage of the reference silicone layer as a sensor of local pressure as described in detail in [55]. To synthesize such a single “standardized OCE image”, a series of initial structural OCT-scans acquired during monotonic compression of the sample was first processed to obtain a series of cumulative-strain maps as described in [54,57,58]. Then vertical A-scans corresponding to the selected pressure were picked up from the initial series of cumulative-strain maps and reassembled to synthesize a single cumulative-strain image in which all A-scans now correspond to the same preselected pressure onto the tissue [55]. To be sure that the strain in silicone can be considered linearly proportional to stress (pressure), high linearity of silicone was specially verified as described in [42,54,55]. Real biological tissues usually demonstrate a pronouncedly nonlinear stress–strain law. The described C-OCE method allows one to determine this law by plotting the strain in the linear precalibrated silicone against strain in any region of interest in the tissue beneath the silicone. The elasticity of the tissue can then be estimated as the tangent Young’s modulus (the slope of the stress-strain curve) corresponding to the desired pre-selected pressure. It was empirically found that for breast-cancer tissue the sought tangent modulus could be conveniently estimated as the slope of the chorde corresponding to the pressure range 4 ± 1 kPa. At lower pressures, very small strain of stiffer regions was difficult to estimate, whereas at higher pressures, the elasticity contrast among various tumor components became worse because of strong nonlinearity-induced stiffening of the initially softer components of the tumor (see examples in [55]). Without such standardization the intrinsic elastic nonlinearity of breast-cancer tissues may result in uncontrollable variability of the estimated elastic modulus in different measurements and even different parts of the same image. This unpredictable variability may be rather significant (several times and greater) even for apparently moderate strains within a few percent [42,55]. Thus, the developed pressure-standardization procedures were critically important for enabling meaningful quantitative comparisons of elastographic data obtained from different measurements.

The so-obtained OCE-images were represented in the color-coded form, such that stiffer areas (those with weaker strain) are shown in blue, and soft areas, where deformation is greater, are shown in red.

### 2.3. Histological Study

After CP-OCT and C-OCE imaging of the freshly-excised sample with yet non-modified optical and biomechanical properties, the scanned area was marked on the specimen with histological ink. Then the specimen was fixed in 10% formalin for 48 h and resectioned through the marked area, so that the plane of the histological sections coincided to the cross-sectional CP-OCT and C-OCE images. For the histological evaluation, haematoxylin and eosin (H&E) staining was used. Two independent histopathologists interpreted the histological slices photographed in transmitted light with a Leica DM2500 DFC (Leica Microsystems, Wetzlar, Germany) microscope, equipped with a digital camera. Based on histopathological analysis, all samples were classified into tumorous and non-tumorous breast tissues. The revealed histological types of breast tissue include: adipose tissue with streaks of connective tissue (number of specimens *n* = 20); fibroadenomatosis/fibroadenoma (*n* = 4); DCIS (*n* = 5); invasive lobular carcinoma (ILC) (*n* = 7); invasive ductal carcinoma (IDC) of scirrhous (*n* = 24) and solid (*n* = 10) structure (Table 1). In addition, to assess tumor aggressiveness (prognosis of the disease course) immunohistochemistry (for *n* = 46 samples) was performed, identifying five molecular subtypes of the tumors: Luminal A, Luminal B (Her2/Neo-), Her2/Neo+, Non-luminal, Triple-negative cancer (TNC). Luminal A and Luminal B (Her2/Neo-) are reported to be low-aggressive tumors characterized by predominantly favorable prognosis of disease course and treatment in comparison with Her2/Neo+, Non-luminal and TNC [24]. Furthermore, it should be noted that Luminal A and Luminal B subtypes were characterized by scirrhous architectonics, while Her2/Neo+, Non-luminal, TNC had solid structure.

The results of histopathology were compared with the corresponding CP-OCT-based and C-OCE-based findings. For the blinded reader study, all images were divided into 4 groups: adipose and normal stromal breast tissue, benign breast tissue (fibroadenoma/fibroadenomatosis), non-invasive DCIS, and images portraying cancerous features of invasive low and highly-aggressive breast cancer.

### 2.4. Reader Analysis of CP-OCT and C-OCE Images

A blinded reader study was performed to evaluate the statistical performance of assessing tumorous and non-tumorous breast tissues based on the CP-OCT imaging (first test) and C-OCE visualization (second test). In the study, 115 CP-OCT and 115 C-OCE images from 50 patients were interpreted by 6 readers specially trained for this OCT-based assessment (2 biologists experienced in optical imaging, but unskilled in recognizing breast cancer pathology; 2 post-graduate students of the Medical University unexperienced both in optical imaging and in recognizing breast cancer pathology; 2 surgeons skilled in detecting breast cancer pathology, but without work experience in optical imaging) who were unaware whether the image contained cancer or not. The readers were given a training set of sample CP-OCT and C-OCE images (3 images of each histological type of breast tissue).

The criteria evaluated by the readers are summarized in Table 2 and Table 3. Each image group had its own set of visual criteria. The reader’s goal was to distinguish between tumorous and non-tumorous breast tissues. If an image was considered to represent non-tumorous breast tissue, the reader indicated a score of “0” whether it was normal breast tissue or fibroadenoma. If the reader identified malignant lesion marks, a score from “1” to “3” was assigned to the sample depending on the estimated tumor aggressiveness. The score of “1” means that the reader thinks that the image represents non-invasive DCIS; a score of “2” means that the reader considers the cancer to be invasive, but less aggressive; a score of “3” means that the reader thinks that invasive cancer is more aggressive.

The first test was based on assessment of signal architecture in cross- polarization images (Table 2). The cross-polarization channel enables more contrast visualization of the presence and state of connective tissue in comparison with the co-polarization OCT images.

**Figure 1:**
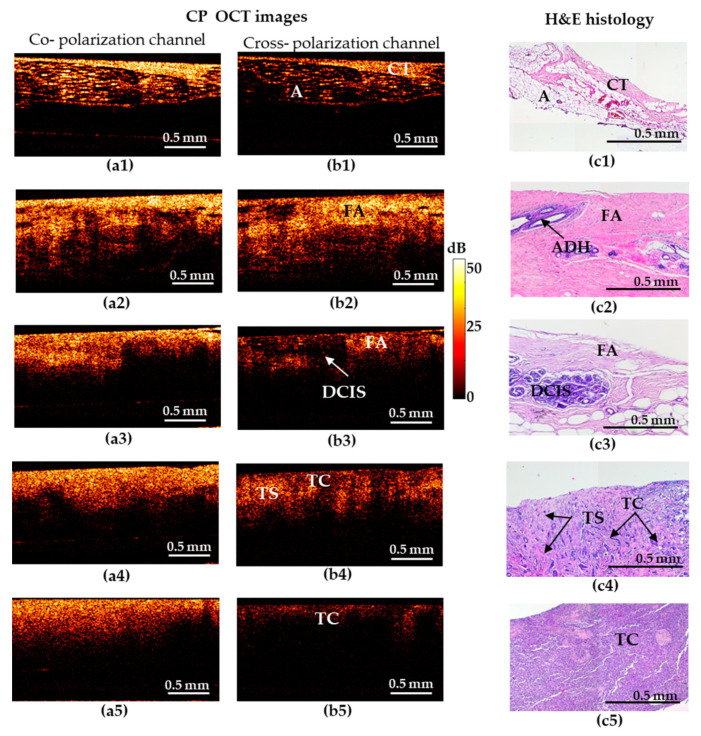
Representative depth-wise co- and cross-polarization OCT images (**a**,**b**) of non-tumorous and tumorous breast tissue with the corresponding histology (**c**). (**a1**–**c1**) Adipose tissue with streaks of connective tissue; (**a2**–**c2**) fibroadenomatosis/fibroadenoma; (**a3**–**c3**) DCIS; (**a4**–**c4**) invasive ductal carcinoma (IDC) of scirrhous structure (low-aggressive breast cancer subtype); (**a5**–**c5**) IDC of solid structure (highly-aggressive breast cancer subtype). (**a1**–**a5**) OCT images in co-polarization channel; (**b1**–**b5**) OCT images in cross-polarization channel; (**c1**–**c5**) histological images, haematoxylin and eosin (H&E) staining. Abbreviations: A—adipose, CT—connective tissue, FA—fibroadenomatosis, ADH—atypical ductal hyperplasia, DCIS—ductal carcinoma in situ, TS—tumor stroma, TC—cluster of tumor cells.

Structural features in the CP-OCT images were distinguished by the following features of the scattering intensity and lateral uniformity of the signal attenuation (Table 2):

(i) the average level of the CP-OCT signal throughout the image is visually estimated as “low” like in Figure 1(b5) or “high” for the used 0–50 dB signal range, where “low” corresponded to intensities below 25 dB, i.e., the noise range in the used scale, and “high” related to the level above 25 dB on the used scale like in Figure 1(b2);

(ii) the presence of structures with a sharp boundary between contrasting-in-brightness regions with well-circumscribed boundary architecture like in Figure 1(b3) (which was graded as “yes”/“no”);

(iii) the attenuation rate as estimated by the penetration depth of the probing radiation (“high” attenuation like in Figure 1(b5) and “low” like in Figure 1(b2));

(iv) the uniformity of attenuation along the interior border of the structural CP-OCT image (“uniform” like in Figure 1(b2)/“non-uniform” like in Figure 1(b4)).

The second test was based on the analysis of stiffness values distributions on C-OCE images. Stiffness maps are presented in a color palette, where hard areas (blue—above 500 kPa) indicate the presence of tumor cells, and soft regions (red—below 100 kPa) represent adipose and connective tissues. At the same time, tissues with intermediate stiffness (the predominance of orange and yellow colors corresponding to ~200–400 kPa) correspond to the presence of such degenerative changes of breast-tissue stroma as fibrosis or hyalinosis of collagen fibers. The threshold values for stiffness (Table 3) of the main types of breast-tissue components were identified as described in detail in our previous work [25], in which accurate comparison of histological and OCE images was performed.

Main and additional criteria of subsuming the images to one or another group were formulated for cross-polarization and C-OCE images, the additional criteria of visual assessment being needed for more precise differentiation among different malignant states (Table 2 and Table 3).

### 2.5. Statistical Analysis

The results of the blinded reader study of CP-OCT and C-OCE images analysis were collected for determining the diagnostic accuracy for distinguishing: (1) non-tumorous breast tissues (*n* = 33) from tumor (*n* = 82); (2) non-invasive DCIS (*n* = 10)) from invasive breast cancer (*n* = 72); (3) low-aggressive invasive tumors (Luminal A, Luminal B (Her2/Neo-)) (*n* = 47) with favorable prognosis from highly aggressive invasive tumors (Her2/Neo+, Non-luminal, TNC) (*n* = 25) with unfavorable prognosis; (4) non-tumorous breast tissues (*n* = 33) from low-aggressive invasive tumors (*n* = 47), and (5) non-tumorous breast tissues (*n* = 33) from highly-aggressive invasive tumors (*n* = 25).

The statistical analysis was performed using Statistica 10.0 and IBM SPSS Statistics software.

The assessment of the informative value and diagnostic capabilities of the studied methods (CP-OCT and C-OCE) was carried out with an estimation of their sensitivity (Se), specificity (Sp), and diagnostic accuracy (Ac). Based on the sensitivity and specificity values, Receiver operating characteristic (ROC) curves were constructed, which show the dependence of the number of true positive rate (TP) on the number of false positive rate (FN). For quantitative characterization of the ROC curves, we evaluated the area under the ROC curve (AUC), i.e.**,** the area bounded by the ROC curve and the axis of the false positive rate [59]. The higher the AUC, the better the classifier is.

The inter-reader agreement was calculated using Cohen’s kappa coefficient (k): k ≥ 0.81—perfect agreement; 0.61 ≤ k < 0.80—substantial agreement; k < 0.6—poor agreement [60].

## 3. Results

### 3.1. Visual Assessment of the CP-OCT and C-OCE Images for Distinguishing between Non-Tumorous and Tumorous Breast Tissue

**Figure 2:**
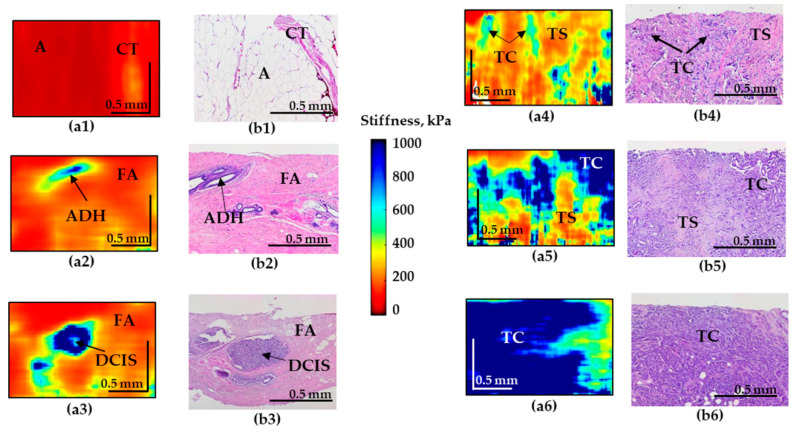
Representative depth-wise C-OCE images (**a1**–**a6**) of non-tumorous and tumorous breast tissue with corresponding histological images (**b1**–**b6**). (**a1**–**b1**) Adipose tissue with streaks of connective tissue; (**a2**–**b2**) fibroadenomatosis/fibroadenoma; (**a3**–**b3**) DCIS; (**a4**,**a5**–**b4**,**b5**) IDC of scirrhous structure (low-aggressive breast cancer subtypes); (**a6**–**b6**) IDC of solid structure (highly-aggressive breast cancer subtype). Abbreviations: A—adipose, CT—connective tissue, ADH—atypical ductal hyperplasia, FA—fibroadenomatosis, DCIS—ductal carcinoma in situ, TS—tumor stroma, TC—cluster of tumor cells.

The results based on the CP-OCT and C-OCE images for representative cases of the non-tumorous and tumorous breast tissue and differentiation among highly-aggressive breast-cancer subtypes are shown in Figure 1 and Figure 2.

Figure 1 shows five types of representative CP-OCT and histological images: “adipose tissue with streaks of connective tissue” (a1–c1)/”fibroadenomatosis/fibroadenoma” (a2–c2)/“DCIS” (a3–c3)/invasive low-aggressive breast cancer of scirrhous structure (a4–c4)/invasive highly-aggressive breast cancer of solid structure (a5–c5).

Benign breast tissue states are characterized by high signal-penetration depth and uniformity of the signal attenuation along the inferior border in co- and cross-polarized structural OCT images (Figure 1). The hallmark of normal adipose (fatty) tissue is a “honeycomb” structure with low sparse scattering, while fibrous structures are characterized by high uniform scattering in co- and cross-polarized structural OCT images (Figure 1(a1–c1)). Fibroadenoma is characterized by a predominance of high-intensity OCT signal in co- and cross-polarization channels (Figure 1(a2–c2)) in comparison with normal breast tissue that has a dense structure due to the presence of large fibrous collagen fibers (Figure 1(c2)).

Cases suspicious for malignancy are characterized by general reduction in signal intensity and its penetration depth, irregular inferior border. All these features cause heterogeneity of the image. In particular, DCIS (Figure 1(c3)) is characterized by the presence of localized structures with low signal intensity and clear boundaries in the surrounding fibrous stroma with a high signal intensity in the cross-polarization channel (Figure 1(b3)). In co-polarization channels DCIS is not detectable (Figure 1(a3)).

In case of invasive breast cancer, the OCT signal in the cross-polarization channel for highly-aggressive (Figure 1(b5)) and less-aggressive (Figure 1(b4)) cancer subtypes is greatly different. IDC of solid structure (highly-aggressive) demonstrates a uniform low-level OCT signal, which is associated with an increased density of tumor cells and an almost total absence of anisotropic (fibrous) structures in this tumor subtype (Figure 1(b5)). For IDC of scirrhous structure (less-aggressive subtype), the heterogeneity of the OCT signal was observed: an alternating signal of medium and low intensity was revealed (Figure 1(b4)). On the corresponding histological images, there were clusters of tumor cells surrounded by connective tissue in a state of fibrosis and hyalinosis (Figure 1(c4)), which clearly leads to an increase in the level of OCT signal in these areas. It should be noted that in these cases, there is no pronounced contrast between low-aggressive (Figure 1(a4)) and highly-aggressive (Figure 1(a5)) breast cancer subtypes in the co-polarization channel.

Thus, in the structural OCT images, the most informative is the cross- polarization channel showing both regions with fairly high cross-polarization backscattering and (corresponding to the presence of connective tissue) and regions with a reduced cross-polarization signal (corresponding to the clusters of tumor cells), see Figure 1(b1–b5)). Therefore, in view of low informativity of the co-polarization images, only cross- polarization images were used for diagnostic accuracy analysis in this study.

The C-OCE image of the normal mammary gland (normal connective tissue and adipose tissue) is characterized by the lowest stiffness (Figure 2(a1)). However, fibroadenomatosis/fibroadenoma is characterized by a slight overall increase in stiffness (Figure 2(a2)) and the presence of well-localized areas with an increased elastic modulus in the regions of atypical ductal hyperplasia (ADH).

C-OCE images of malignancy demonstrate the appearance of regions with pronouncedly increased stiffness. Moreover, for IDC of solid structure (highly-aggressive), these areas occupy up to 90% of the entire image, which sharply distinguishes this breast cancer subtype (Figure 2(a6)). The ducts filled with tumor cells for DCIS are visualized as high-contrast zones with strongly increased stiffness (Figure 2(a3)) which coincide well with the histological image. The surrounding fibrous tissue is characterized by fairly low stiffness values (Figure 2(a3)). The OCE images of IDC of scirrhous structure demonstrate an increased stiffness in the regions of the clusters of tumor cells and significantly lower stiffness in the regions of the tumor stroma, causing multiple moderately contrast inclusions with elevated stiffness, which represents a feature of low-aggressive tumor subtype (Figure 2(a4–a5)).

In addition, it is necessary to mention that images of IDC of scirrhous structure and fibroadenoma may have similar patterns that may be challenging to differentiate for the reader. To solve this problem an additional criteria (Table 3) of “presence the numerous and less contrasting inclusions of increased stiffness” was included in cases of IDC (Figure 2(a4)) in contrast to single inclusions in cases of fibroadenoma (Figure 2(a2)) and DCIS (Figure 2(a3)).

### 3.2. Diagnostic Accuracy of CP-OCT and C-OCE Based on Visual Assessment of Images

The results of the two tests, using the identified main and additional criteria, separately in CP-OCT images and C-OCE images demonstrate their great agreement among the readers. The concordance coefficient in the determination of tumorous or non-tumorous breast tissue in the analysis of CP-OCT images between two researchers was k = 0.68, between two post-graduate students k = 0.93, between the two surgeons k = 0.80. The concordance coefficient in the detection of tissue type in the analysis of C-OCE images between two researchers was k = 0.86, between two post-graduate students k = 0.93, between the two surgeons k = 0.82.

**Figure 3:**
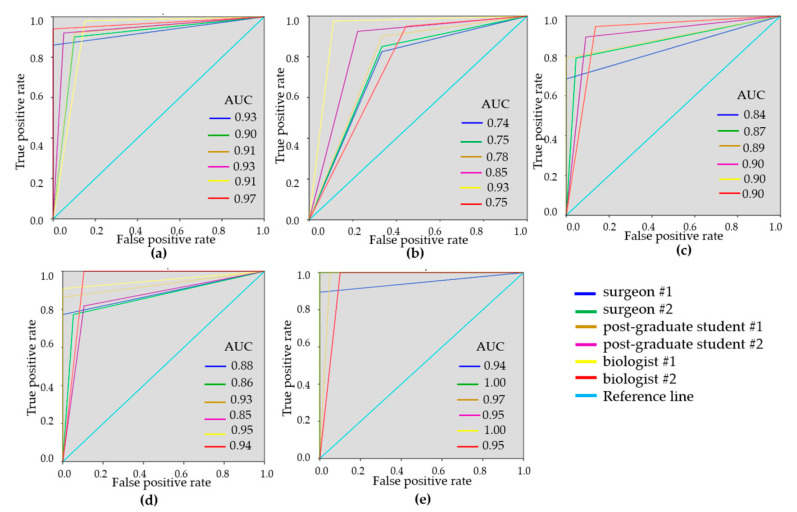
Receiver operating characteristic (ROC)-curves showing the results of visual assessment CP-OCT images for distinguishing non-tumorous breast tissue from tumor (**a**), DCIS from invasive breast cancer (**b**), low-aggressive invasive breast cancer from highly aggressive (**c**), non-tumorous breast tissue from low-aggressive breast cancer (**d**), non-tumorous breast tissue from highly aggressive breast cancer (**e**) for six “blinded” readers.

**Figure 4:**
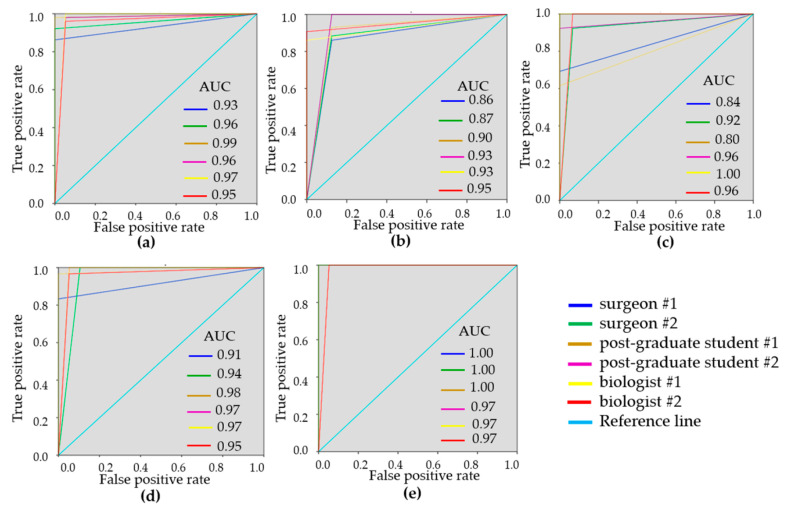
ROC-curves showing the results of visual assessment of C-OCE images for distinguishing non-tumorous from tumorous breast tissue (**a**), DCIS from invasive breast cancer (**b**), low-aggressive invasive breast cancer from highly-aggressive (**c**), non-tumorous breast tissue from low-aggressive breast cancer (**d**), non-tumorous breast tissue from highly-aggressive breast cancer (**e**) for six “blinded” readers.

To demonstrate the variability of the test results, ROC-curves were presented for each reader (Figure 3 and Figure 4). ROC-curves analysis confirmed that visual assessment of CP-OCT and C-OCE images has a high diagnostic value for differentiating non-tumorous and tumorous breast tissue (AUC values for all readers were 0.90–0.97 and 0.93–0.99, respectively) and also for distinguishing between low- and highly-aggressive invasive breast-cancer subtypes (AUC values for all readers were 0.84–0.90 and 0.80–1.00, respectively) (Figure 3c, Figure 4c). Slightly lower values were obtained for differentiation between non-invasive breast lession and invasive breast cancer (AUC values for all readers were 0.74–0.93 and 0.86–0.95, respectively) (Figure 3b, Figure 4b). The ROC-curves show that the best results were demonstrated by the researches experienced in optical imaging.

The results of the blinded reader analysis are summarized in Table 4, showing the sensitivity, specificity and diagnostic accuracy. Each diagnostic index was averaged among all six readers. High diagnostic values were obtained for the differential diagnosis of all analyzed groups. The diagnostic accuracy of distinguishing non-tumorous tissue from tumor was 92.4 ± 2.3% for CP-OCT and 96.0 ± 3.3% for OCE, which determines the OCE method as more specific for detecting tumorous tissue.

For the first time, the diagnostic efficiency of CP-OCT and C-OCE methods for the differential diagnosis of non-invasive from invasive breast cancer was established (Se = 90.1 ± 5.7%, Sp = 70.6 ± 11.3%, Ac = 82.5 ± 7.1% and Se = 90.5 ± 5.3%, Sp = 92.0 ± 6.1%, Ac = 90.4 ± 2.7%, respectively). Furthermore, we demonstrated the possibility to differentiate invasive low-aggressive breast cancer subtypez with a favorable prognosis from highly-aggressive breast cancer subtypes with a poor prognosis for treatment and the course of the disease (Se-83.5 ± 10.5%, Sp-93.5 ± 6.0%, Ac-87.8 ± 6.5% and Se-87.3 ± 13.8 ± 6.5%, Sp-98.0 ± 3.1%, Ac-89.5 ± 10.0%, respectively). In both cases, it was demonstrated that C-OCE showed the best diagnostic indicators (Table 4).

Additionally, we performed a diagnostic analysis of the possibility to distinguish non-tumorous breast tissue from low- and highly-aggressive breast cancer subtypes. It has been shown that the diagnostic accuracy of the difference between non-tumorous breast tissue and a low-aggressive subtype of cancer is 88.1 ± 6.0% for CP-OCT and 95.7 ± 4.1% for C-OCE. The diagnostic accuracy of the difference between non-tumorous breast tissue and highly-aggressive cancer is 97.2 ± 2.8% for CP-OCT and for C-OCE—98.3 ± 2.2%.

Thus, we demonstrated the possibility to use CP-OCT and C-OCE methods for detecting different breast cancer subtypes on the resection margin which would minimize the risk of recurrence and reoperations.

### 3.3. Assessment of Human Breast Cancer Margins

**Figure 5:**
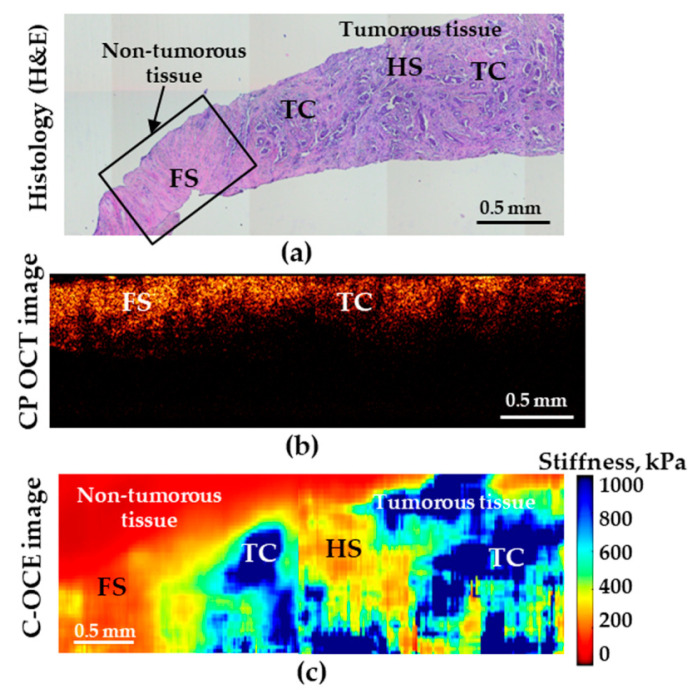
Histological image (**a**) demonstrating transition between non-tumorous (fibrous stroma—FS) and tumorous breast tissues (low-aggressive IDC of scirrhous structure); (**b**) is the corresponding CP-OCT image in the cross-polarization channel and (**c**) is the C-OCE images of the same area. HS denotes hyalinized stroma, and TC—clusters of tumor cells.

The tests performed in this study demonstrated that, in distinguishing the norm from low-aggressive cancers (and, moreover, highly aggressive ones), the analysis of both CP-OCT and C-OCE images the both methods enable high diagnostic accuracy. However, when searching for the transition between IDC of scirrhous structure and non-cancerous tissue, the C-OCE-based stiffness mapping (Figure 5c) visualizes the tumor margin much more clearly in comparison with the cross-polarization images (Figure 5b).

## 4. Discussion

The results presented here show the high diagnostic value and efficiency of CP-OCT and C-OCE methods for differential diagnosis of non-tumorous and tumorous breast tissue, with the further prospect of intraoperative determination of the “positive” margin of tumor resection during breast-conserving surgery in real time. In addition, the diagnostic efficiency of CP-OCT and C-OCE methods for differentiation between non-invasive and invasive breast cancers, as well as between invasive low-aggressive breast cancer subtype with a favorable prognosis (Luminal A, Luminal B (Her2/Neo-)) and highly aggressive breast cancer subtypes with a poor prognosis for course of the disease (Her2/Neo+, Non-luminal, TNC).

In previous studies, only standard visual imaging criteria, such as signal intensity and high/low stiffness, were used for differentiation between tumorous and non-tumorous breast tissues. In this study, additional analysis criteria were proposed, which made it possible to increase the diagnostic sensitivity and specificity, significantly reducing the number of erroneous diagnoses. We identified such additional analysis criteria as the presence of structures and the characteristics of signal attenuation in depth on cross-polarization images, as well as the presence of inclusions and mosaic structure on C-OCE images with visually feasible assessment of several stiffness grades.

Previous works have demonstrated that conventional, intensity-based OCT can provide differentiation between tumorous and non-tumorous breast tissues through both quantitative [18,61,62,63] and qualitative [32,61,63] assessment of the OCT signal. Several studies demonstrated that OCE has the high potential to delineate tumor in breast tissue based on elevated elasticity on a microscale [33,40,41,44]. A recent study [16] demonstrated the ability of structural OCT to identify positive margins in specimens from BCS. The qualitative assessment of OCT images showed the high diagnostic accuracy of structural OCT for distinguishing normal and cancerous tissue within the resection bed following wide local excision of the human breast: sensitivity of 91.7% and specificity of 92.1% [16]. Additionally, visual assessment of C-OCE images for evaluation of tumor margins in specimens excised during breast-conserving surgery also provides high sensitivity of (92.9%) and specificity (96.4%) [43].

Breast cancer is a highly heterogeneous disease, both morphologically and genetically. The surgical approach and the amount of resection depend on the subtype of breast cancer, which, as this study has shown, can be determined in rapid OCT-based tests, including the possibility of intraoperative use. The C-OCE and CP-OCT images provide additional contrast between tumor and normal tissue in comparison with structural OCT. C-OCE and CP-OCT analysis of excised tissue specimens can distinguish between normal and cancerous tissues by identifying the heterogeneous and disorganized connective tissue structures indicative for malignancy. We have demonstrated that differences in the microstructural features of cross-polarization and stiffness images enable differentiation between highly and low-aggressive breast cancer subtypes confirmed by histopathology. For this purpose, the main and additional criteria for assigning an image to a particular group were formulated, which are necessary for a more accurate differentiation of malignant conditions among themselves. For example, a uniform low-intensity in CP-OCT images and a uniform high level of stiffness in C-OCE images characterize tumor of a solid structure, while tumor tissue of a scirrhous structure in the immediate vicinity of non-tumorous breast tissue can also retain homogeneity, or it can lose it and may be represented by different levels of signal intensity and stiffness.

ROC curves were constructed as a measure of overall accuracy for each reader when non-cancerous tissue was distinguished from tumor by CP-OCT and C-OCE methods (Figure 3). In this case, C-OCE showed higher specificity (97.5 ± 2.7% vs. 93.3 ± 6.0%) and diagnostic accuracy (96.0 ± 3.3% vs. 92.4 ± 2.3%) compared to cross-polarized images. This fact may be caused by the difficulty in interpreting qualitative OCT criteria based on signal intensity by readers, in comparison with the criteria for interpreting quantitative OCE images that usually have more contrast and visually easier assessable differences. Overall, for differentiation between tumorous and non-tumorous tissues, the C-OCE method has proved to be more efficient.

Additionally, for more specific differentiation between non-invasive breast cancer and invasive breast cancer, the following diagnostic parameters were determined for CP-OCT and C-OCE methods: Se = 90.1 ± 5.7%, Sp = 70.6 ± 11.3%, Ac = 82.5 ± 7.1% and Se = 90.5 ± 5.3%, Sp = 92.0 ± 6.1%, Ac = 90.4 ± 2.7%, respectively. For distinguishing between invasive low-aggressive and highly-aggressive breast cancer subtypes, the CP-OCT and C-OCE gave the following results: Se = 83.5 ± 10.5%, Sp = 93.5 ± 6.0%, Ac = 87.8 ± 6.5% and Se = 87.3 ± 13.8 ± 6.5%, Sp = 98.0 ± 3.1%, Ac = 89.5 ± 10.0%, respectively. Therefore, in both cases, C-OCE showed better diagnostic indicators (Table 4).

The diagnostic accuracy of the difference between non-tumorous breast tissue and low-aggressive breast cancer for CP-OCT and C-OCE was found to be fairly high, 88.1 ± 6.0% and 95.7 ± 4.1%, respectively. Even higher was the Ac of CP-OCT and C-OCE for the difference between non-tumorous breast tissue and highly-aggressive breast cancer (97.2 ± 2.8% and 98.3 ± 2.2%, respectively).

Accurate determining of the boundaries of tumor resection is more feasible for tumors of a solid structure in comparison with tumors of scirrhous structure that may resemble fibroadenomas in OCT-based images. However, the performed targeted histological examination has given a clue for better understanding of the causes of stiffness increase or decrease and made it possible to define additional criteria that improved the diagnostic accuracy of C-OCE for various breast cancer subtypes detection, including non-invasive and low-aggressive tumors.

Thus, the formulated additional (clarifying) criteria for visual assessment of CP-OCT and C-OCE images provided a higher diagnostic accuracy in differentiation between tumorous and non-tumorous breast tissues with various grades of aggressiveness. In the future, this will increase the value of these OCT-based methods in detecting the boundaries of tumor resection during BCS.

## 5. Conclusions

Both CP-OCT and C-OCE data may be helpful to a surgeon–oncologist for more accurate detection of a “clean” resection margin during breast-conserving surgery. The test based on assessment of C-OCE images has shown higher diagnostic accuracy (96%) and sensitivity (95%) in comparison with CP-OCT images (Se—92%, Ac—92.4%) for breast cancer detection. Furthermore, the preformed study demonstrated high potential of CP-OCT and C-OCE for differentiating particular molecular-biological and morphological subtypes of breast cancer with assessment of the tumor aggressiveness, which is important for subsequent treatment selection.

## Figures and Tables

**Table 1:** Clinical specimens’ characteristics and number of imaged specimens.

Diagnosis	Number of Specimens	Age of Patients (Range)	Tumor Size
**Benign breast conditions**			
Non-tumorous breast tissue	20	43–68	-
Fibroadenoma/fibroadenomatosis	4	35–48	≤1 cm
**Malignant breast lesions**			
Ductal carcinoma in situ (DCIS)	5	44–63	≤1 cm
Invasive ductal carcinoma (IDC) of scirrhous structure	24	41–82	≤2 cm
Invasive ductal carcinoma (IDC) of solid structure	10	41–82	≤2 cm
Invasive lobular carcinoma (ILC) of solid structure	7	48–72	≤2 cm

**Table 2:** Visual assessment criteria of cross-polarization optical coherence tomography (CP-OCT) images for distinguishing between non-tumorous and tumorous breast tissue.

	Normal (Non-Tumorous) Breast Tissue (*n* = 20)	Fibroadenoma/Fibroadenomatosis (*n* = 13)	DCIS (*n* = 10)	Low-Aggressive Invasive Breast Cancer (*n* = 47)	Highly-Aggressive Invasive Breast Cancer (*n* = 25)
**Main criterion:**					
Typical architecture	honeycomb structure, areas of high intensity signal	predominance of areas with high signal intensity	alternating signal of high, medium and low intensity; the presence of structures with no signal with clear boundaries (ducts)	alternating signal of medium and low intensity	homogenous low intensity signal
**Additional criteria:**					
Signal penetration depth	high	high	high	high	low
Structures with clear (contrasting) boundaries	no	no	yes	no	no
Uniformity of the OCT signal attenuation along the inferior border of the image	uniform	uniform	highly uneven	non-uniform	uniform
Final score	0	0	1	2	3

*n*—number of images.

**Table 3:** Visual assessment criteria of compressional optical coherence elastography (C-OCE) images for distinguishing between non-tumorous and tumorous breast tissue.

	Normal (Non-Tumorous) Breast Tissue (*n* = 20)	Fibroadenoma/Fibroadenomatosis (*n* = 13)	DCIS (*n* = 10)	Low-Aggressive Invasive Breast Cancer (*n* = 47)	Highly-Aggressive Invasive Breast Cancer (*n* = 25)
**Main criterion:**					
Typical stiffness pattern	uniform low stiffness level over the C-OCE image		low stiffness level of the stiffness throughout the image with high-contrast zones with strongly increased stiffness	non-uniform high stiffness level over the C-OCE image	
**Additional criteria:**					
Predominance of uniform distribution of high stiffness values (>500 kPa)	no	no	no	no	yes
Presence of multiple moderately contrast inclusions of high stiffness (Mosaic structure)	no	no	no	yes	no
Final score	0	0	1	2	3

*n*—number of images.

**Table 4:** The results of diagnostic test for visual assessment of the CP-OCT and C-OCE images.

	AUC (Range)	Sensitivity (Se), %	Specificity (Sp), %	Diagnostic Accuracy (Ac), %
**CP** **-** **OCT imaging**				
Non-tumorous versus tumorous breast tissue	0.90–0.97	92.0 ± 4.0	93.3 ± 6.0	92.4 ± 2.3
DCIS versus invasive breast cancer	0.74–0.93	90.1 ± 5.7	70.6 ± 11.3	82.5 ± 7.1
Low-aggressive versus highly-aggressive breast cancer	0.84–0.90	83.5 ± 10.5	93.5 ± 6.0	87.8 ± 6.5
Non-tumorous breast tissue versus low-aggressive breast cancer	0.85–0.95	85.1 ± 8.8	95.8 ± 4.9	88.1 ± 6.0
Non-tumorous breast tissue versus highly-aggressive breast cancer	0.94–1.00	98.1 ± 4.4	95.8 ± 4.9	97.2 ± 2.8
**C-OCE imaging**				
Non-tumorous versus tumorous breast tissue	0.93–0.99	95.0 ± 5.1	97.5 ± 2.7	96.0 ± 3.3
DCIS versus invasive breast cancer	0.86–0.95	90.5 ± 5.3	92.0 ± 6.1	90.4 ± 2.7
Low-aggressive versus highly-aggressive breast cancer	0.80–1.00	87.3 ± 13.8	98.0 ± 3.1	89.5 ± 10.0
Non-tumorous breast tissue versus low-aggressive breast cancer	0.91–0.98	95.8 ± 6.5	95.8 ± 3.7	95.7 ± 4.1
Non-tumorous breast tissue versus highly-aggressive breast cancer	0.97–1.00	98.6 ± 3.2	97.5 ± 2.7	98.3 ± 2.2
